# Clinical value of pulmonary congestion detection by lung ultrasound in patients with chronic heart failure

**DOI:** 10.1002/clc.23738

**Published:** 2021-10-01

**Authors:** Na Li, Yunlong Zhu, Jianping Zeng

**Affiliations:** ^1^ Department of Cardiology Xiangtan Central Hospital Xiangtan China; ^2^ Center of Cooperative Postgraduate Cultivation in Xiangtan Central Hospital University of South China Xiangtan China

**Keywords:** chronic heart failure, lung ultrasound, pulmonary congestion

## Abstract

Chronic heart failure is one of the common causes of hospitalization and death. Pulmonary congestion is the common disease feature of patients with chronic heart failure, which could be correctly diagnosed by lung ultrasound. Efficacy of lung ultrasound‐guided pulmonary congestion management for patients with acute heart failure is well documented, however, more evidence is needed to establish the clinical value of pulmonary congestion detection by lung ultrasound examination in patients with chronic heart failure. This review summarized current evidence related to the use and clinical value of pulmonary congestion assessment by lung ultrasound in patients with chronic heart failure, aiming to provide new suggestions on promoting the widespread use of lung ultrasound in patients with chronic heart failure to improve the quality of life and outcome of patients with chronic heart failure.

## 1 INTRODUCTION

Chronic heart failure (CHF) is a clinical syndrome with various etiologies and associated with multiple comorbidities, frequent hospitalization, reduced quality of life and high mortality.^1^ Mortality was about 2%–17% during the first admission with heat failure, 17%–45% at 1 year, and >50% within 5 years.^2^ Heart failure resulted in considerable and growing economic burden on the health care systems.^3^ Mortality of patients with acute myocardial infarction and myocarditis is significantly reduced due to effective therapy options during the last decade.^4^, ^5^ However, survived patients with residual myocardial injury might gradually develop CHF and contribute to the increased prevalence of CHF. Population aging serves as another important reason for the increased prevalence of CHF. Despite advances in diagnosis and treatment of CHF, the 5 years mortality of CHF remained as high as around 50%.^4^ Given the irreversible nature of heart failure, it is of importance to take additional efforts to reduce the adverse impact of CHF on health care system and to improve the quality of life and outcome of CHF patients.^6^

Pulmonary congestion (PC) is a common disease feature and associated with poor outcome in patients with heart failure, including CHF.^7^ According to the results of a two‐center cohort study, about 23% of heart failure patients were discharged with residual PC and residual PC at discharge was related to poor outcome.^8^ Timely monitoring and effectively relieving of PC might thus serve as an important strategy of heart failure management during hospitalization and post hospital discharge.

Clinically, PC could be evaluated with multiple approaches.^9^ Physicians may palpate the jugular pulse and auscultate the lung rales to estimate PC, but the sensitivity of these signs is not satisfactory.^10^ Chest X‐rays could be used to detect PC by observing radiographic signs of fluid accumulation in the lung interstitium or alveolar space, but bedside X‐ray equipment, which would be an optimal tool for the examination of CHF patients, is not always available in daily clinical practice setting. PC could be properly assessed by measuring pulmonary capillary wedge pressure (PCWP) with right heart catheterization, but the invasive feature of this procedure limits its widespread use in the daily clinical practice.^11^ Lung ultrasound (LUS) is another semi‐quantitative method for the evaluation of PC. LUS could detect PC at bedside, the sensibility and specificity of LUS on detecting PC are 46% and 95%, respectively. Gullett et al.^12^ evaluated agreement among trained emergency physicians assessing the degree of B‐line presence on bedside ultrasound in patients presenting to the emergency department (ED) with acute undifferentiated dyspnea. They also determined which thoracic zones offered the highest level of interobserver reliability for sonographic B‐line assessment. The right and left anterior/superior lung zones showed substantial agreement in all assessment methods and demonstrated best overall agreement (ICC for right: counting, ordinal, and normal/abnormal, 0.811 [0.714–0.875], 0.875 [0.810–0.917], and 0.729 [0.590–0.821], respectively). Furthermore, both expert/expert pairs and expert/novice pairs showed substantial agreement in the right and left anterior/superior thoracic zones (expert/expert, 0.904 and 0.777, respectively; expert/novice, 0.862, and 0.834, respectively). Interrater agreement was best in the anterior/superior thoracic zones followed by the lateral/superior zones for both expert/expert and expert/novice pairs. Agreement in the lateral/inferior lung zones was overall inferior. Intrarater agreement was highest at extreme high or low numbers of B‐lines. Table 1 summarized the advantages and limitations of LUS, CT, and right heart catheterization on the evaluation of PC.

**TABLE 1:** Contrast the auxiliary examination instrument for pulmonary congestion

	Lung ultrasound	CT	RH catheterization
Advantages	Non‐invasive	Non‐invasive	Precision
	No‐radiation	Objective	No‐radiation
	Cost effective		
	Time‐saving		
Disadvantages	Semi‐quantitative evaluation	Radiation	Invasive
Features	Detecting the B‐line	Signs of fluid accumulation in the lung interstitium or alveolar space	Measurement of pulmonary capillary wedge pressure
Sensitivity (%)	46	44	82
Specificity (%)	95	94	57

Abbreviations: CT, computed tomography; RH catheterization, right heart catheterization.

LUS has been increasingly used in clinical practice for monitoring the status of PC nowadays, especially in patients with acute heart failure.^11^, ^13^, ^14^ Evidence related to PC monitoring and the impact of LUS‐guided PC management in CHF patients is also accumulating now. Based on obtained clinical experience with the use of LUS, several guidelines and expert consensus recommended the use of LUS in the clinical setting (Table 2). It is to note more randomized large cohort clinical trials are needed to enhance the recommendation level of applying this examination technique in daily clinical practice. Exacerbation of PC could be evaluated by multiple modalities, including invasive PICCO, Swan‐Ganz pulmonary artery catheters, implantable devices (CardioMEMS™ system, OptiVol™ Fluid Status Monitoring) and non‐invasive pulmonary resistance assessment equipment, chest X‐ray, chest CT, echocardiography, LUS and wearable health equipment), which are reviewed in detail by Bekfani^15^ and Bashi.^16^ Among them, LUS serves as the simplest and most timesaving non‐invasive method for the sequential monitoring of PC changes. This point of view is gaining more and more acceptance recently.^17^, ^18^ We recommend the use of the 6‐point and 8‐point methods for the assessment of PC, LUS detected B lines ≥3 could be used as a reliable cutoff value,^19^ patients with LUS detected B lines ≥3 are highly suggestive of PC.^20^

**TABLE 2:** Guideline and expert consensus recommended clinical application of lung ultrasound

Guidelines	Year	Contents
ESC guideline^17^	2016	Thoracic ultrasound may be considered for the confirmation of pulmonary congestion and pleural effusion in patients with HF (Class IIb; Level C).
JCS 2017/JHFS 2017 Guideline^18^	2017	Lung ultrasound assessment has been reported to be beneficial in the diagnosis of pulmonary edema.
Expert consensus document^23^	2019	Lung ultrasound is a useful tool for the assessment of patients with both acute and chronic heart failure.
The Heart Failure Association of the European Society of Cardiology^45^	2020	During the first hours of admission, the point‐of‐care focused cardiac and lung ultrasound examination is an invaluable tool for rapid differential diagnosis of acute dyspnea.

Current consensus is that the combination of LUS with already widely used biomarkers, echocardiography and other cardiac imaging modalities might enhance the diagnosis accuracy and contribute to the decision making of more efficient therapeutic strategies in patients with CHF.^17^ This article summarized the current status and future prospective of the clinical use and value of LUS on the assessment of PC in CHF patients based on literature review.

## 2 INCIDENCE AND IMPACT ON OUTCOME OF PULMONARY CONGESTION IN PATIENTS WITH CHRONIC HEART FAILURE

Numerous trials and epidemiological studies have demonstrated the prevalence of PC in patients with CHF.^19^, ^21^, ^22^, ^23^, ^24^ LUS‐HF study is a single‐blind clinical trial, 123 patients admitted for HF were included in this trial,^25^ the post hoc analysis of the LUS‐HF trial showed that that up to 40% of patients considered “dry” according to pulmonary auscultation presented LUS‐evidenced PC at hospital discharge, and these patients also experienced worse prognosis at 6‐month follow‐up.^21^ Platz and colleagues examined 195 NYHA class II–IV HF patients during routine cardiology outpatient visits with LUS, and 185 patients with adequate LUS images in all zones were analyzed, the results showed that prevalence of patients with ≥3 B‐lines on five‐ or eight‐zone LUS was around 32%, and these patients faced about fourfold increased risk of 6‐month HF hospitalization or death.^19^ They also found that PC count was also positively linked with other clinical and laboratory markers of HF.^19^ In a prospective cohort study, Dwyer et al. reported data from outpatient echocardiography and LUS for 111 hypertensive, 46 HFpEF and 73 HFrEF patients, the prevalence of ≥3 B‐lines was 13.5%, 34.8%, and 45.2%, respectively, again, worse outcome was found in HF patients with ≥3 B‐lines (age‐ and sex‐adjusted hazard ratio 2.62, 95% CI 1.15, 5.96; *p* = .022)^24^(Tables 3 and 4). Domingo et al. reported the impact of the number of LUS detected B‐line and outcome in 577 chronic HF stable ambulatory patients, results showed that the total sum of B‐lines remained as an independent predictive factor of the composite endpoint (hazard ratio 1.04, 95% CI 1.02–1.06, *p* = .002) and of all‐cause death (hazard ratio 1.04, 95% CI 1.02–1.07, *p* = .001), independently of whether or not N‐terminal pro‐B‐type natriuretic peptide (NT‐proBNP) was included in the model (*p* = .01 and *p* = .008, respectively), with a 3%–4% increased risk for each 1‐line addition.^26^

**TABLE 3:** Acute heart failure and chronic heart failure related trials

Study (year)	Cohort	*N*	Conclusion
Marini et al (2020)^6^	Outpatients with CHF	244	LUS‐guided management reduces hospitalization for ADHF at mid‐term follow‐up in outpatients with chronic HF.
EPICC Study (2019)^14^	Discharged patients with CHF	152	This study will provide more evidence about the impact of LUS on treatment monitoring in patients with chronic HF.
Domingo et al (2020)^26^	Outpatients with stable CHF	577	B‐lines count is an independent risk factor of death or HF hospitalization.
Gargani et al (2021)^46^	Hospitalized cardiac conditions patients with AHF and non‐AHF	1021	LUS measured B‐lines can detect subclinical pulmonary interstitial edema and provide useful information for the diagnosis and the prognosis. Their added prognostic value among standard echocardiographic parameters is more robust in patients with HFpEF compared with HFrEF.
Pivetta et al (2019)^47^	Hospitalized patients with AHF	518	In adult patients presenting to the emergency department with acute dyspnea, a diagnostic protocol based on the integration of LUS and clinical assessment is more accurate than the currently recommended diagnostic approach based on clinical evaluation, CXR and NT‐pro BNP measurement.
Pang et al (2021)^48^	Hospitalized patients with AHF	130	AHF patients with LUS did not show any significant decrease in PC within 6 h compared with usual standard care.

**TABLE 4:** HFpEF, HFmrEF and HFrEF related trials

Study	*N*	Follow‐up	Main outcome	Conclusion
HFpEF and HFrEF				
Palazzuoli et al.^49^	162	6 months	First occurrence re‐hospitalization for AHF or all‐cause mortality	LUS measure lung congestion at discharge provides prognostic information for patients with either HFpEF or HFrEF.
Yang et al.^50^	82	–	HFpEF&HFrEF associations of B‐lines with E/e', NT‐proBNP	LUS measured B‐lines are positively correlated with E/e' and NT‐proBNP but negatively correlated with EF in both the HFpEF and HFrEF groups. The correlation of B‐lines with E/e' was better, especially in the HFpEF group.
Gargani et al.^46^	1021	14.4 months	Death and rehospitalization for AHF	LUS prognostic value is more robust in patients with HFpEF compared with HFrEF.
Dwyer et al.^24^, ^51^	230	12 months	HF hospitalization or all‐cause mortality	B‐lines is higher in patients with either HFpEF or HFrEF than in hypertensive patients who are at risk for HF.
HFpEF				
Coiro et al.^51^	61	1 year	Cardiovascular death or HF hospitalization	LUS is an independent predictor of adverse outcome in New York Heart Association I/II patients with HFpEF.
Rueda‐Camino et al.^52^	103	3 months	Readmission and death attributable to worsening heart failure	Patients with 15 B‐lines are 2.5 times more likely to be readmitted for acute heart failure than less congestive patients.
HFpEF, HFmrEF and HFrEF				
Pellicori et al.^53^	342	234 days	Composite of all‐cause mortality or heart failure hospitalization	Each clinical and ultrasound measure of congestion including B‐lines was associated with increased risk but, in multivariable models, only higher NT‐proBNP and IVC, and lower JVD ratio, were associated with the composite outcome.
Mozzini et al.^54^	120	18 months	Discharge time	The B‐lines clearance time is longer in patients with HFrEF compared to those with HFpEF and HfmrEF. LUS is useful in tailoring diuretic therapy and speeding up the discharge time in hospitalized HF patients.
Rivas‐Lasarte et al.^25^	123	At 14 days, 1 month, 3 months, and 6 months after hospital discharge	Composite of urgent visits, hospitalization for worsening HF and death from any cause	Tailored LUS‐guided diuretic treatment of pulmonary congestion in this proof‐of‐concept study reduced the number of decompensations and improved walking capacity in patients with HF.

## 3 CLINICAL MANIFESTATION OF PULMONARY CONGESTION IN CHRONIC HEART FAILURE PATIENTS

**FIGURE 1:**
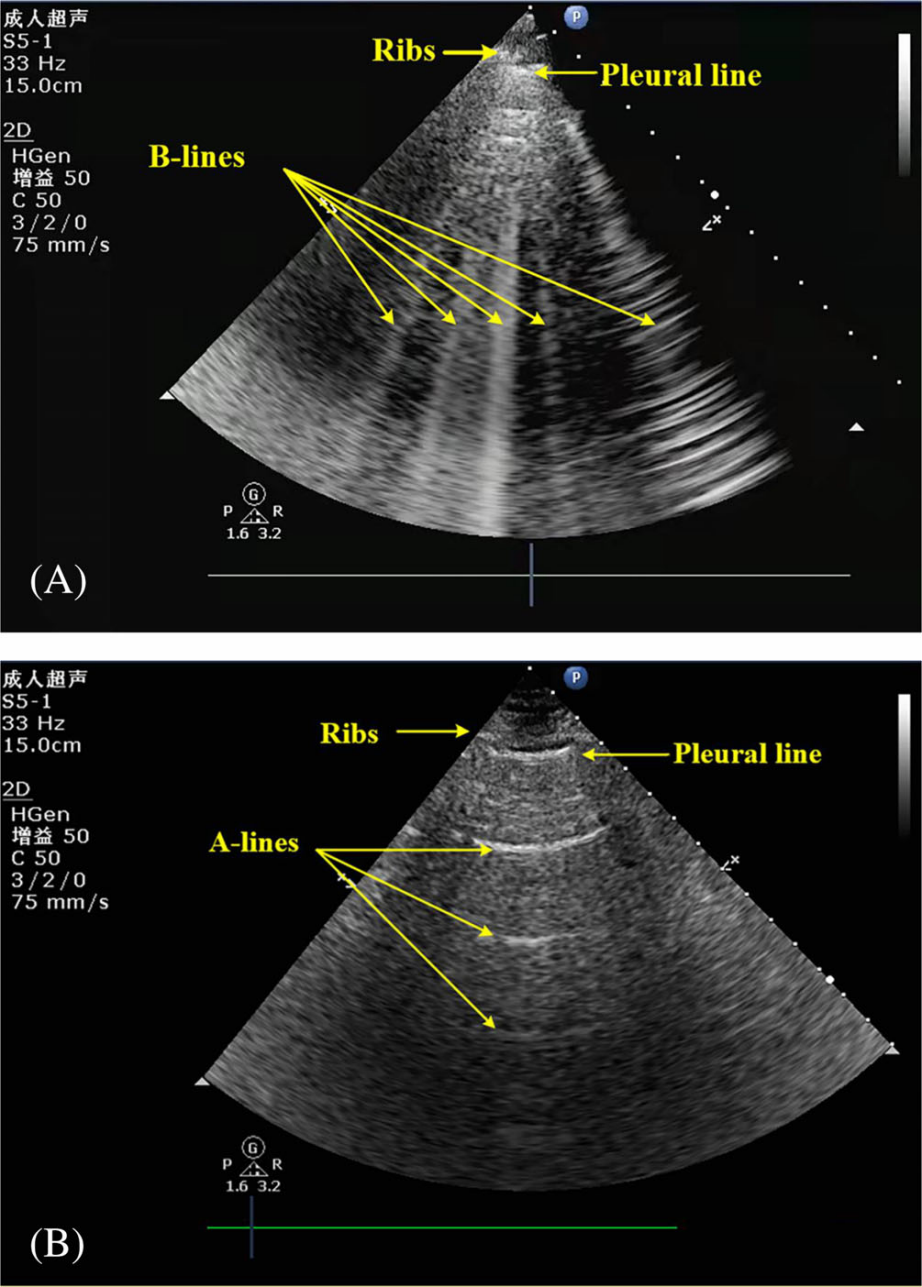
(A) Five B‐lines were counted by 8–point methods in a patient admitted with acute decompensated chronic heart failure. (B) The B‐lines from the same patient disappeared and A‐line was obvious at discharge

Major determinants of pulmonary congestion are inflammation, LV filling pressure, lymphatic drainage, hydrostatic and oncotic pressure.^27^, ^28^ Congestion can manifest as venous systemic congestion and fluid retention. Congestion can affect multiple organs, including lung tissue, liver, kidney, gut, and extremities. Congestion is one of the major drivers of worsening heart failure symptoms.^28^ Pulmonary congestion is mainly induced by elevated LV filling pressure in patients CHF.^28^ Moreover, systemic inflammation can increase vascular permeability and enhance PC. In addition, disturbance in the integrity of the alveolar‐capillary membranes, lymphatic drainage, and increased hydrostatic and oncotic pressure could also result in PC.^27^ The common clinical symptoms of PC are fatigue and dyspnea. Common clinical signs of PC are pulmonary rales, S3, jugular vein distention, peripheral edema, and reduced exercise capacities.^29^, ^30^ Since the clinical symptoms and signs of PC are unspecific. Diagnosis of PC relies on the evidence of LUS‐derived B‐lines (LU‐BL). LU‐BL shapes like a comet‐tail, which the vertical lines are extending from the pleural line to the bottom of the screen^31^ (Figure 1), which can move synchronously with lung sliding.^32^ Impedance of lung gas is high is high in the healthy status, so ultrasound could hardly penetrate the lung field. In case of PC, lung gas and fluids could form an air–liquid interface, which changes acoustic impedance and favors the penetration of ultrasound in the lung field.^33^ The clinical value of detecting LU‐BL in chronic heart failure patients is gaining the attention of physicians worldwide now.^24^

## 4 PULMONARY CONGESTION DIAGNOSIS BY LUNG ULTRASOUND IN PATIENTS

LUS has now been used in the diagnosis of ARDS, pneumonia, pulmonary embolism, pneumothorax, COPD, asthma, and interstitial pulmonary fibrosis. In particular, LUS B‐lines could indicate the presence of pulmonary congestion in patients with pulmonary edema, ARDS, and pneumonia^20^ (Table 5).

**TABLE 5:** Association between various diseases and B‐lines

	Lung ultrasound	B‐lines
Disease	Pulmonary edema/congestion	Symmertric B‐lines
	ARDS	Diffuse distribution B‐lines
	Pneumonia	Diffuse distribution B‐lines
	Pulmonary embolism	No
	Peumothorax	No
	COPD	No
	Asthma	No
	Interstitial pulmonary fibrosis	No

Abbreviations: ARDS, acute respiratory distress syndrome; COPD, chronic obstructive pulmonary disease.

**FIGURE 2:**
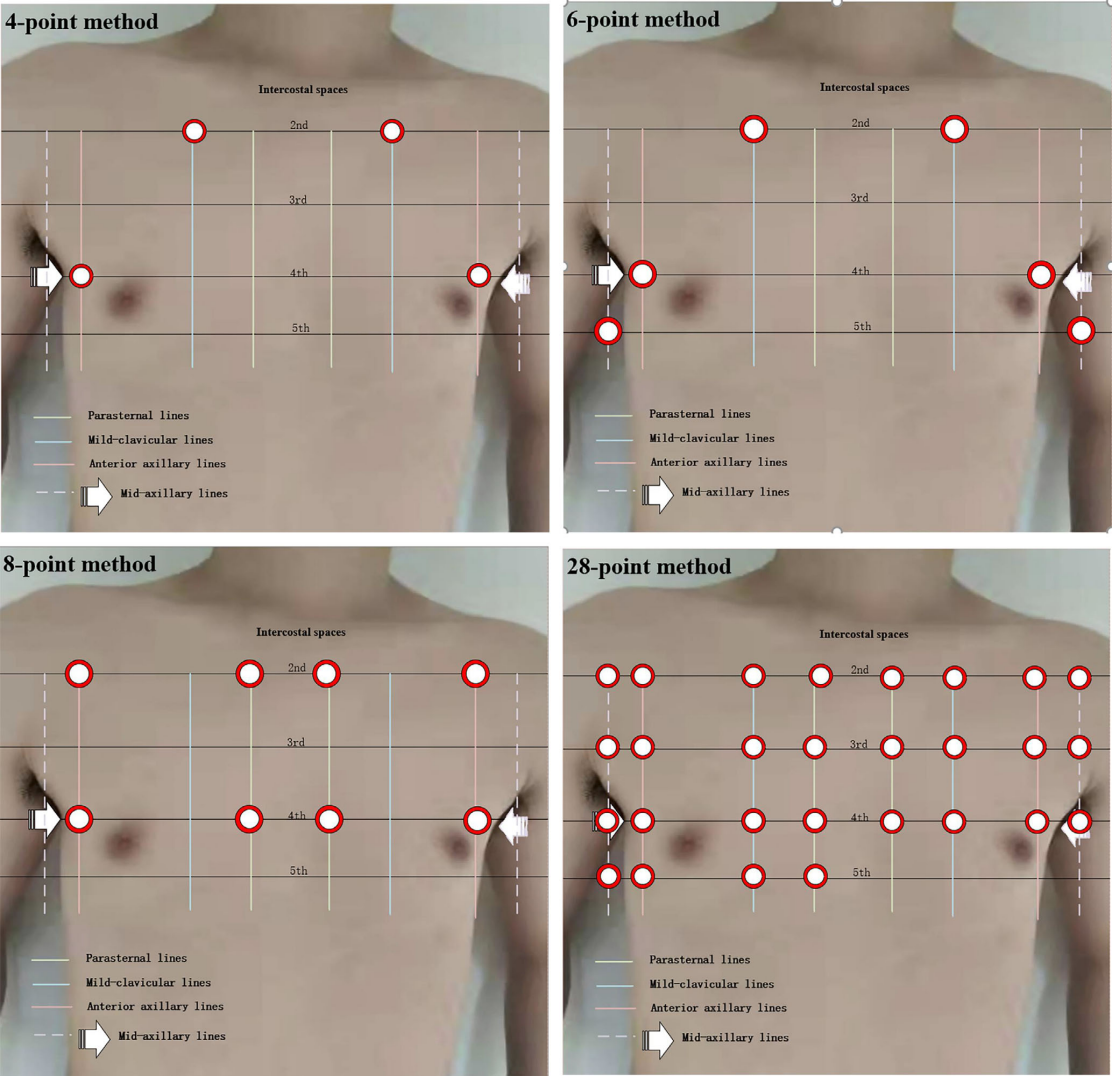
LUS methods of 4‐, 6‐, 8‐, and 28‐point method for the detection of pulmonary congestion by lung ultrasound

LUS is usually performed by qualified researchers with national training experience and certificate. LUS examinations should be performed with the patient in the supine position. The B‐lines could be assessed by 4‐, 6‐, 8‐ or 28‐point methods through scanning the anterior and lateral chest, the transducer should orientate parallel to the ribs (Figure 2). In each intercostal space, the number of B‐lines should be quantified real‐time, counted one by one. Offline image analysis could be performed by video recognition by two investigators with experience in LUS analysis. The LUS area with the largest number of B‐lines should be selected.

The 8‐ and 28‐point methods have been recommended to assess pulmonary interstitial edema.^34^ However, clinical use of the 28‐point method is hampered due to the time‐consuming feature. The 4‐point method to identify CHF may be less effective than the 6‐/8‐point method. The 4‐point method and the‐28 point method each have method‐related advantages. The 4‐point method is simple and fast. It is suitable for crude screening of emergency patients. This is the authoritative method used by the BLUE protocol. Buessler et al.^35^ compared the accuracy of several lung ultrasound methods for the diagnosis of acute heart failure in the ED and found that the 6‐ and 8‐point methods were the most relevant LUS methods for diagnosis of AHF. By including 117 AHF patients, they identified 27.4%, 56.2%,54.8%, and 76.7% of patients with the 4‐, 6‐, 8‐, and 28‐point methods, respectively. Similarly, the C‐index of the 4‐, 6‐, 8‐, and 28‐point methods were 63.7 (58.5–68.8), 72.4 (65.0–79.8), 74.0 (67.1–80.9), and 72.4(63.9–80.9), respectively. CHF patients are likely to exhibit less extensive pulmonary abnormalities than patients admitted in the ICU and may therefore benefit from LUS techniques involving 6‐point methods or more.^35^ Aurélien et al. demonstrated that 6‐/8‐point methods (using the one bilateral positive point threshold) could improve the accuracy of LUS B‐lines in diagnosing AHF.^35^ Eight‐point method is mostly performed for patients in supine position and the procedure time is 3–5 min to complete the examination on an individual patient.^36^ LUS examinations were performed with the patient in the supine position. In each intercostal space, the number of B‐lines was quantified real‐time, and counted one‐by‐one. Offline image analysis could be performed by video recognition by investigators with experience in LUS analysis. The LUS area with the largest number of B‐lines should be selected (Figure 2). A study published in 2015 showed that higher number of B‐lines was identified on the 4‐ versus 2‐second LUS clips (*p* < .001 for 4‐point method, *p* = .001 for 8‐point method).^37^ After personal training and certificate examination of lung ultrasound, qualified individuals can trust their images obtained by portable hand‐held ultrasound probe, to aid their decisions about adjusting medication in individual CHF patient. Platz et al. performed a comparative trial of pocket ultrasound and high‐end ultrasound devices both using in heart failure patients. They included 21 hospitalized patients with heart failure (81% men; median age, 73; 71% Caucasian), who underwent concurrent 8‐ and 4‐point LUS methods. Compared with high‐end ultrasound, the pocket device demonstrated similar sensitivity (89%, 95% CI 68–100% vs. 69%, 95% CI 44–94%), but lower specificity (50%, 95% CI 28–72% vs. 88%, 95% CI 76–100%). As a whole, similar number of detectable B‐lines was reported by both pocket device and high‐end ultrasound system.^37^ Figure 2 showed B‐lines detected in an 84‐year‐old male patient admitted to our department due to acute decompensated chronic heart failure. Five B‐lines were counted by 8‐point methods (Figure 1A). The patient's B‐line disappeared and A‐line was obvious at discharge (Figure 1B).

## 5 IMPACT OF MONITORING AND TARGETING PULMONARY CONGESTION IN CHRONIC HEART FAILURE PATIENTS

LUS offers specific visualization of B‐lines of pulmonary congestion. Theoretically, monitoring PC might be helpful for the adjustment of the dosage of medication, especially the diuretics in CHF patients. Another merit of PC assessment with LUS is to avoid stopping diuretic treatment in superficial stable CHF patients with residual PC. Clinical evidence is accumulating on the value of LUS‐guided PC monitoring the improvement of outcome in patients with chronic heart failure now. There was no causal relationship between PC and ultrasound A‐lines and B‐Lines of the lung, so the number of A‐lines and gray zones does not need to be considered when adjusting medications.^38^ LUS could semi‐quantitatively quantify the B‐lines, as a sign of pulmonary congestion.

Rivas‐Lasarte et al. conducted a single‐blind clinical trial, 123 patients admitted for HF were randomized to either a standard follow‐up (*n* = 62, control group) or a LUS‐guided follow‐up (*n* = 61, LUS group). Tailored LUS‐guided diuretic treatment of pulmonary congestion in this proof‐of‐concept study reduced the number of decompensations and improved walking capacity in patients with HF.^25^ In another single‐blinded, randomized controlled trial, CHF patients were randomized into the LUS‐guided arm or control arm (*n* = 63 each). Patients were followed in four prespecified visits during a 6‐month period, LUS‐guided treatment was associated with a 45% risk reduction in the primary end point (hazard ratio 0.55, 95% CI 0.31–0.98, *p* = .044), mainly driven by a reduction in urgent HF visits (hazard ratio 0.28, 95% CI 0.13–0.62, *p* = .001), while no significant differences in prehospitalization for HF or death were found.^39^ In a randomized multi‐center unblinded study, patients with chronic HF and optimized medical therapy were randomized into ‘physical examination + LUS’ group and ‘physical examination only’ group. Diuretic therapy was modified according to LUS findings and physical examination. Results showed that LUS‐guided management reduced hospitalization for ADHF at mid‐term follow‐up in outpatients with chronic HF.^7^ It should be noted that patients enrolled in above studies were relative small and future studies with larger patient cohort are warranted to validate the reported beneficial effects of LUS‐guided HF therapy strategy. The newly published BLUSHED –AHF study reported the results of a multicenter, single blind, ED‐based, pilot trial aiming to determine whether a 6‐h lung ultrasound (LUS)‐guided strategy‐of‐care improves pulmonary congestion over usual management in the emergency department (ED) setting. The secondary goal was to explore whether early targeted intervention leads to improved outcomes. A total of 130 patients were included in this study, although faster resolution of congestion was achieved during the initial 48 h, emergency department use of LUS to target PC conferred no benefit compared with usual care in reducing the number of B‐lines at 6 h or in 30 days alive and out of hospital.

## 6 FUTURE PROSPECTIVE

Obviously, randomized clinical trials with large patient cohort are needed to validate the exact role of PC‐guided therapy on long‐term outcome for CHF patients. PC evaluation by LUS should be included in the regular follow‐up of CHF patients to improve the individual medication and improve outcome of CHF patients. Digital advance should be more actively added to the PC assessment by LUS. Nowadays, a smartphone app is available to support the teaching on 3D/4D obstetrical ultrasound applications in medical education.^40^ Such an app is wished to be developed for lung ultrasound as a teaching software. At this stage, there is an increasing variety of small ultrasound probes in the market, especially portable probes.^41^, ^42^, ^43^, ^44^ For instance, Philips' lumify probe is both a probe and an ultrasound host, which requires only the connection with an Android smart device, either a tablet or a cell phone. It is instantly accessible for ultrasound scanning, which does not require a dedicated monitor for image display. The wave probe from vulva health (CA, USA) could present real‐time images using either a Wi‐Fi‐connected iOS or Android smartphone or an app on a laptop computer. Both techniques could be used for lung sonography in our mind. Patients or family members of patients can then learn and perform the lung ultrasound examination by the app and transmit the ultrasound data directly to the attending physicians to guide the medical care of the patients at home. Such a process might change the way of lung ultrasound guided CHF therapy at home and might potentially improve the quality of life and outcome of CHF patients.

## 7 CONCLUSIONS

Pulmonary congestion is common in patients with CHF, even in stable CHF patients and LUS is a valuable assessment tool for PC with high sensitivity and should be readily applied to CHF patients on top of standardized clinical examinations. Application of LUS‐guided PC management might improve the diagnosis, monitoring and management of patients with CHF. More randomized clinical trials are needed to validate the role of LUS‐guided CHF management for improving the clinical outcome of CHF patients.

## Supporting information


**Table S1**: 4‐, 6‐, 8‐, and 28‐point methods characteristic

## Data Availability

Data sharing is not applicable to this article as all data are presented or analyzed in this study.
