# Supplementary material for: Clinical value of pulmonary congestion detection by lung ultrasound in patients with chronic heart failure
Source: Clin Cardiol. 2021 Oct 1;44(11):1488–96. doi: 10.1002/clc.23738 (PMC8571545; doi:10.1002/clc.23738)
Supplement: Supplementary file 1 — Table S1: 4‐, 6‐, 8‐, and 28‐point methods characteristic [file CLC-44-1488-s001.docx]

**Figure 1 :LUS methods: 4-, 6-, 8-, and 28-point method**


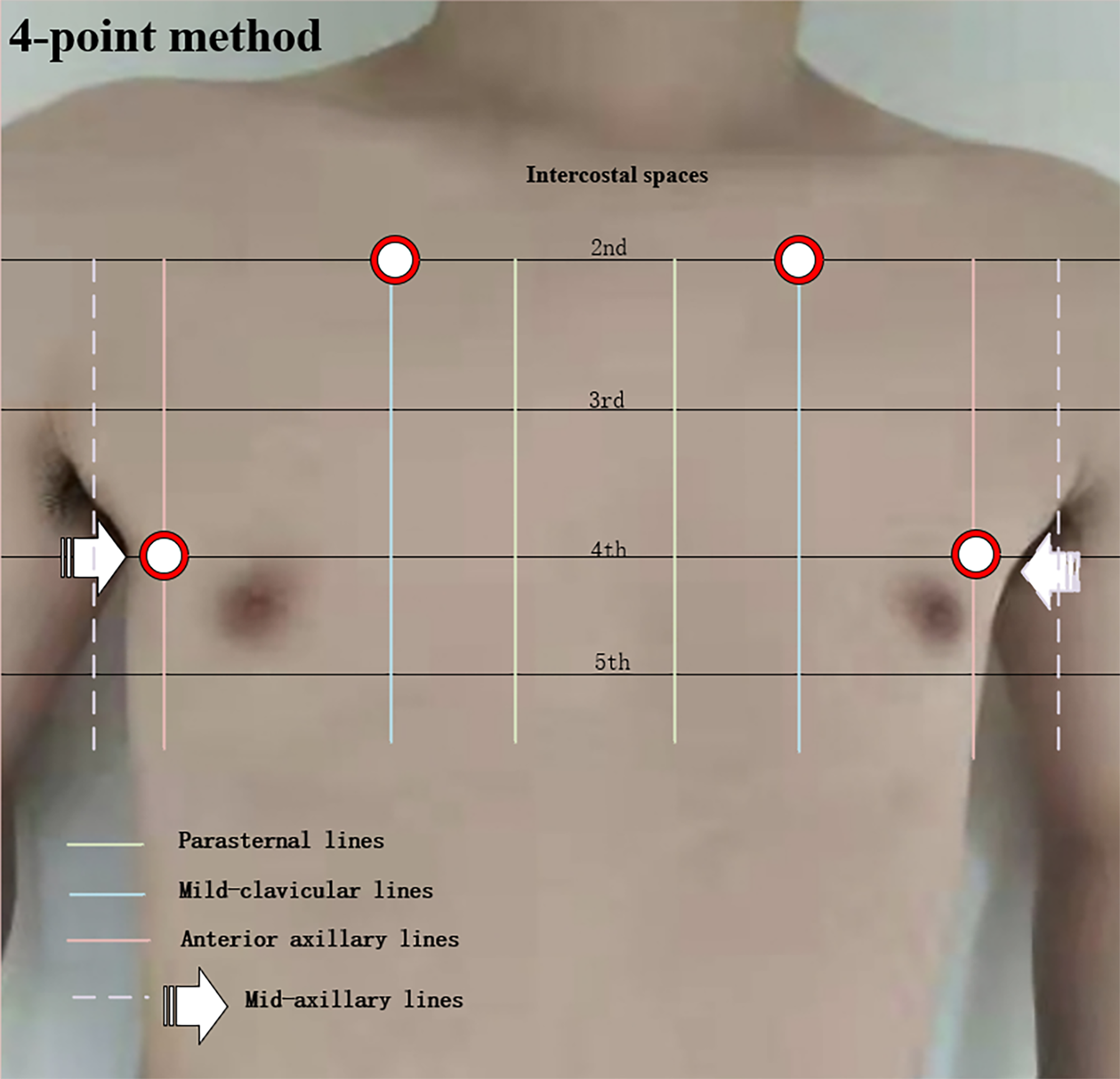

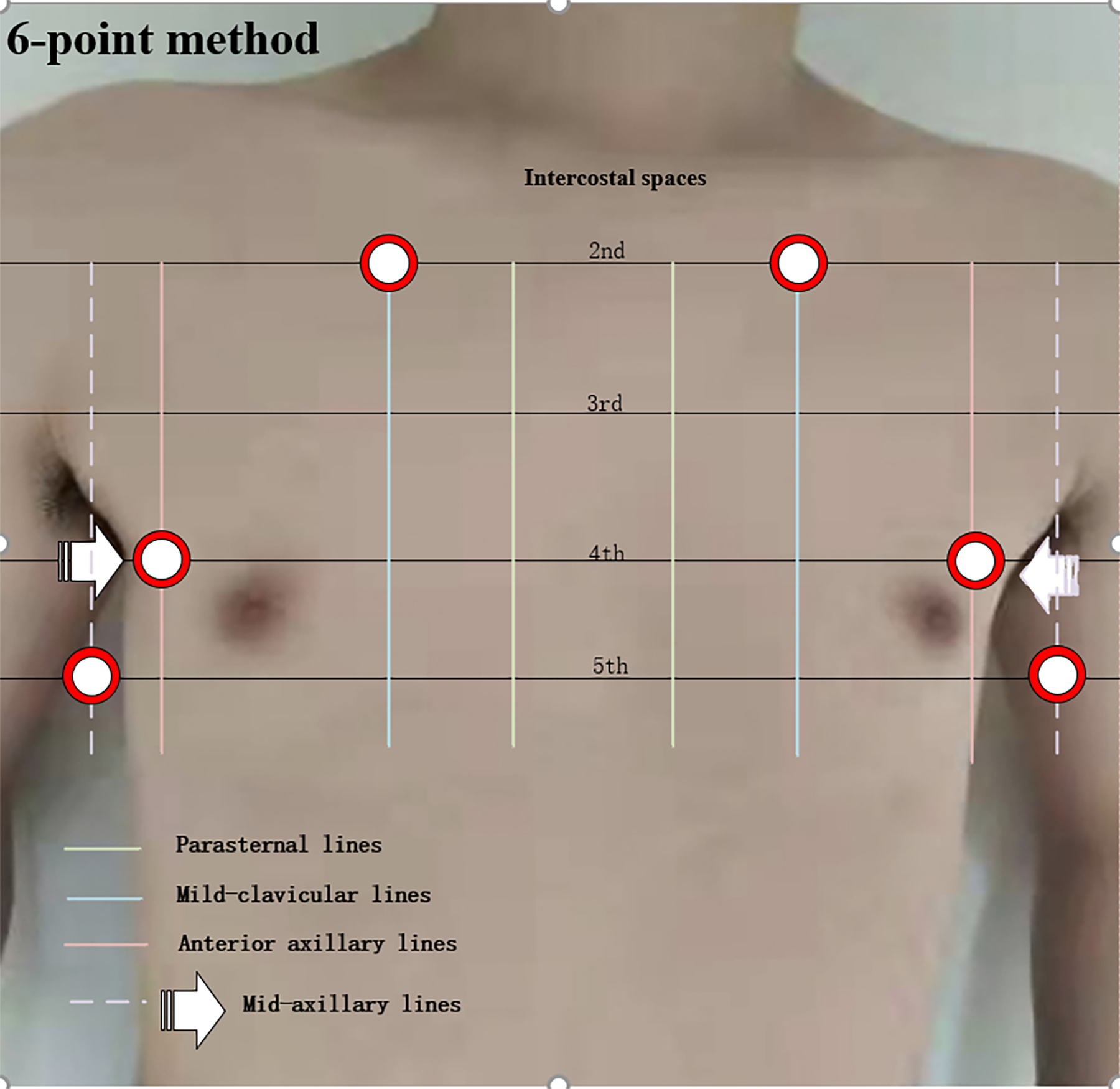

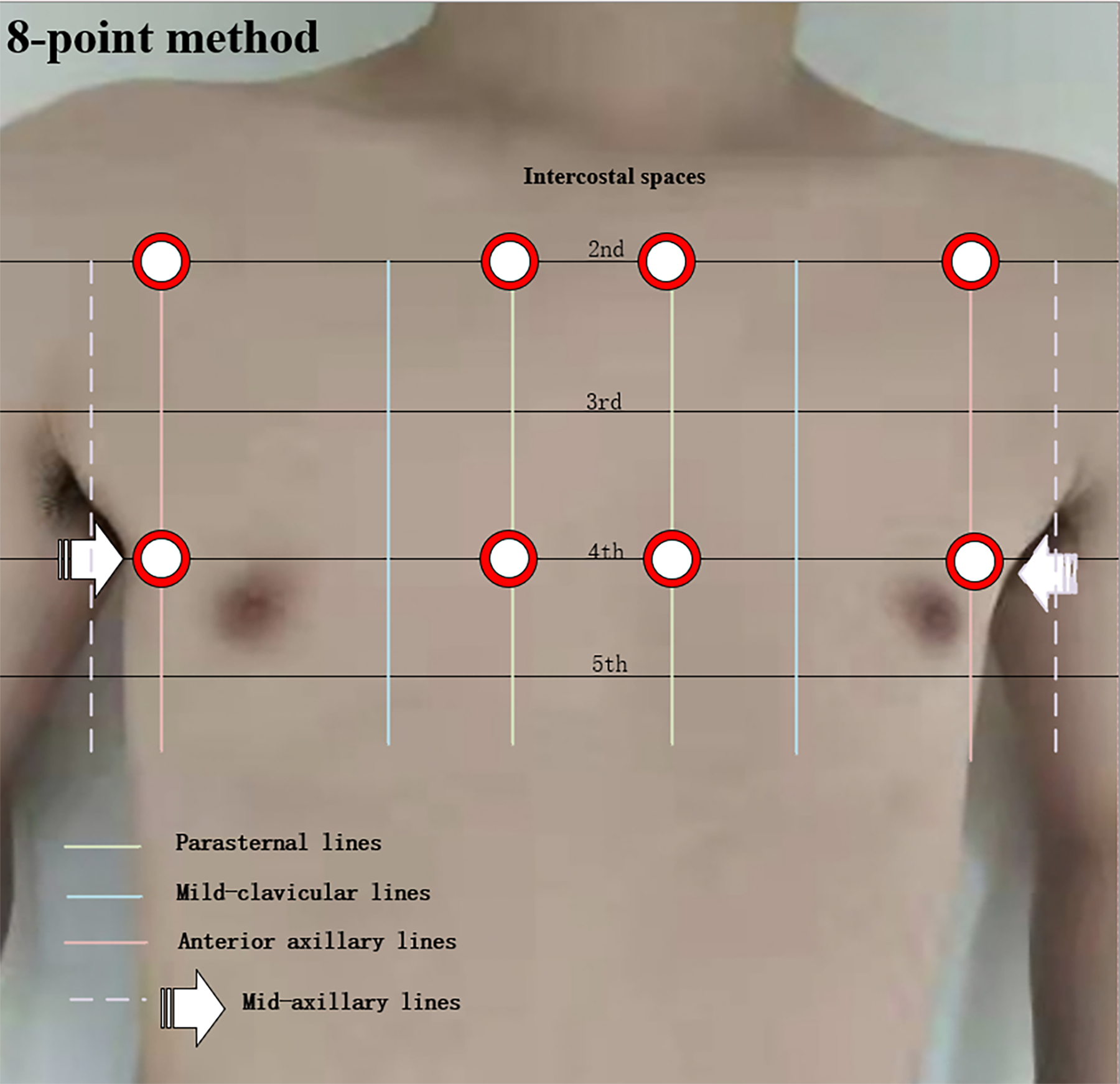

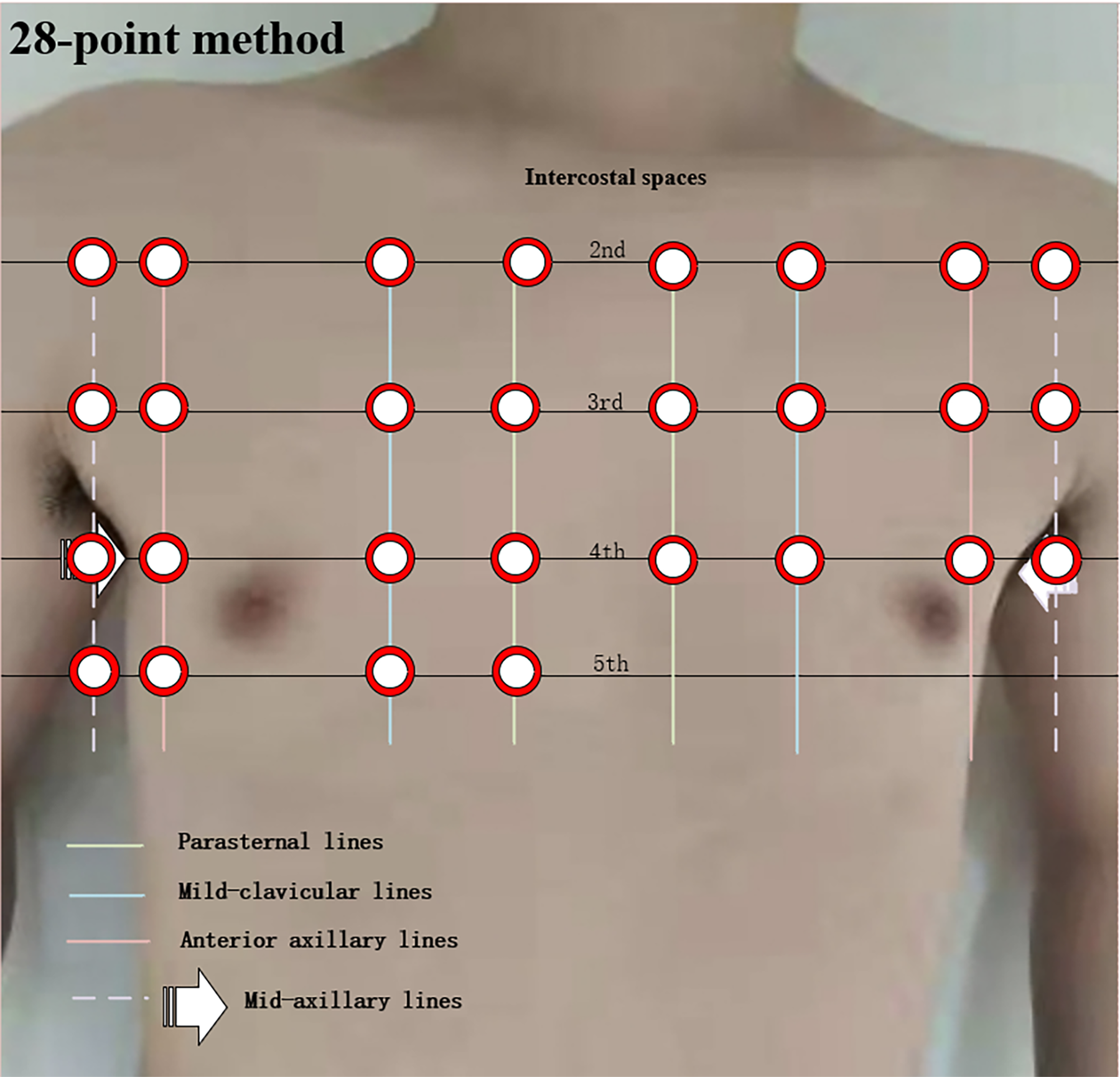


**Figure 2：B-lines of acute decompensated chronic heart failure admission and discharge contrast**


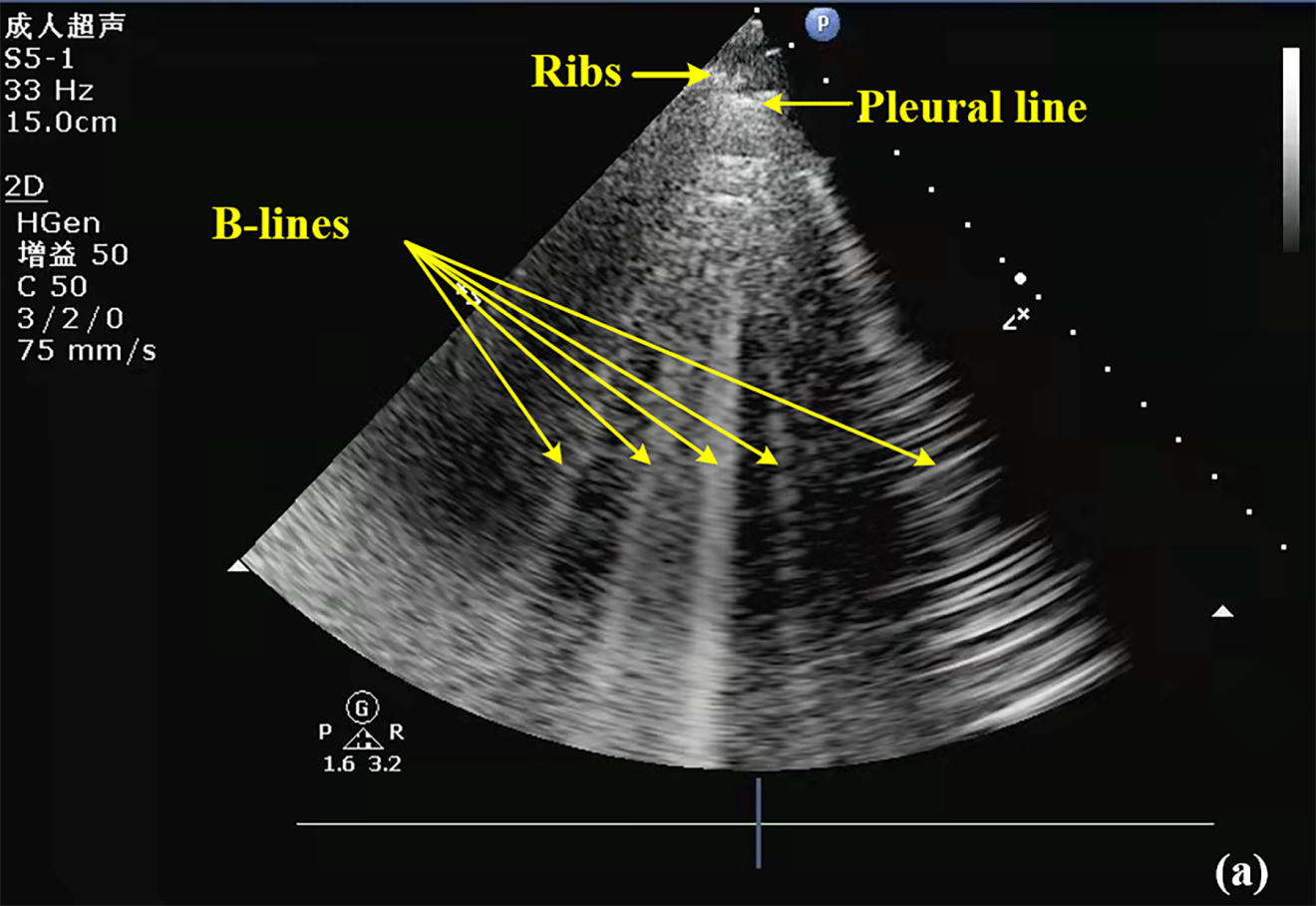

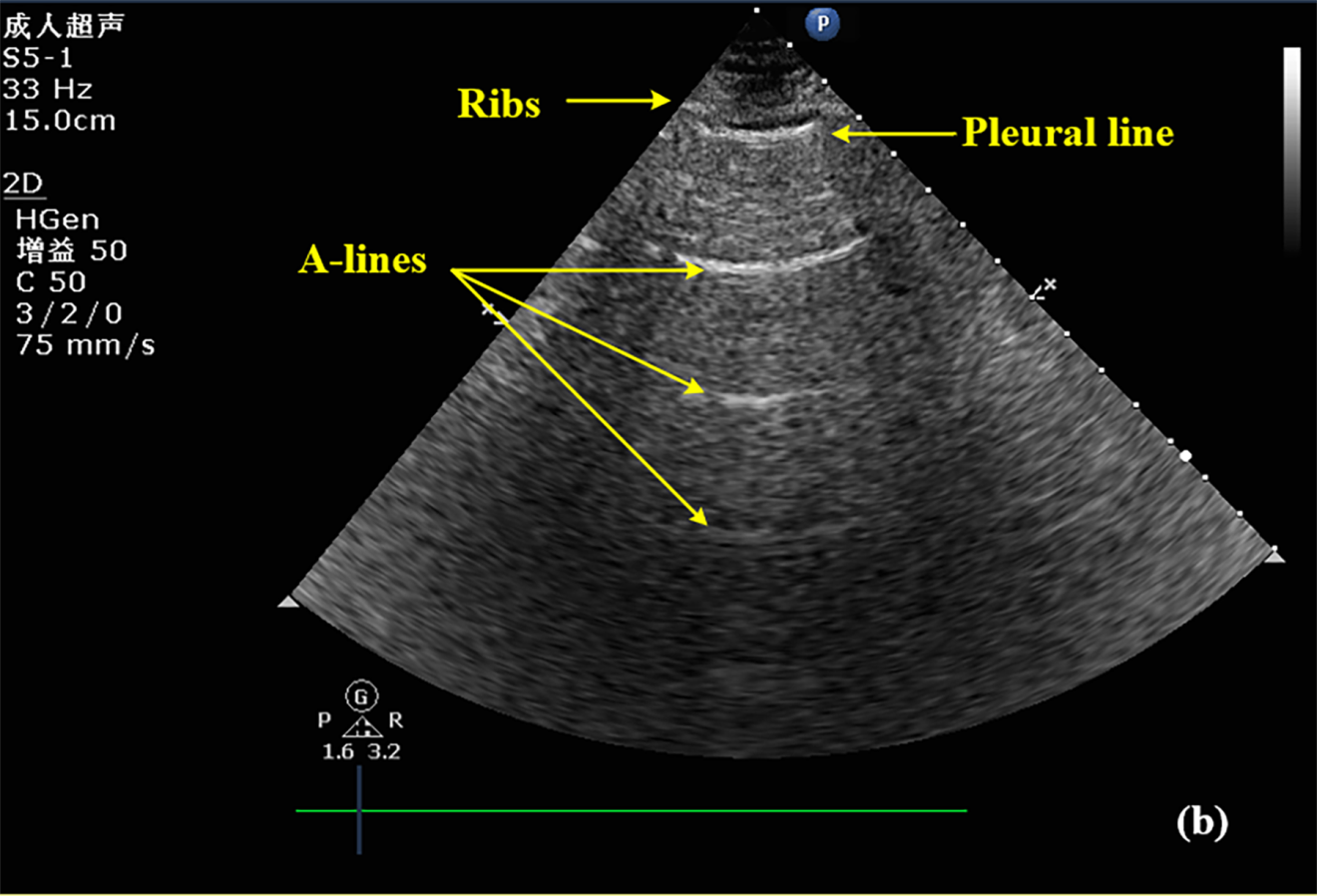


**Supplementary table 1: 4-，6-，8-，28-point methods characteristic**

| **Methods** | **Protocols** | **Position** | **Advantages** | **Brest score Cindex (95%)** |
| --- | --- | --- | --- | --- |
| **28-point** | Miglioranza^9^  Pirompanich^55^  Reisinger^56^ | B-line count ≥30 | 28-point methods are more accurate than 4- and 8-point methods in severe sepsis and ARDS. | 72.4  (63.9-80.9) |
| **8-point** | Platz^19^  Buessler^35^  Iwakura^57^  Reisinger^56^ | ≥ 1 bilateral positive | the most discriminative LUS tools for identifying AHF in elderly patients (mean age, 79.6 years)  more rapid 8- or 6-zone scan is sufficient for the diagnosis of heart failure | 74.0  (67.1-80.9) |
| **6-point** | Pivetta^58^  Buessler^35^  Iwakura^57^  Reisinger^56^ | ≥ 1 bilateral positive | the most discriminative LUS tools for identifying AHF in elderly patients (mean age, 79.6 years)  more rapid 8- or 6-zone scan is sufficient for the diagnosis of heart failure | 72.4  (65.0-79.8) |
| **4-point** | Öhman^59^  Lichtenstein^60^  Iwakura^57^  Platz^61^  Reisinger^56^ | two bilateral positive | the most early and widely used and taught LUS technique  used for the critical patients | 63.7  (58.5-68.8) |
